# Evaluating Trends in Fertility Counseling among Reproductive-Age Patients with Breast Cancer

**DOI:** 10.1245/s10434-025-17857-x

**Published:** 2025-07-24

**Authors:** Sarah Yuen, Katherine McCool, Maryam Bharucha, Kathleen Narloch, Jenny Zhu, Holly Yong

**Affiliations:** Department of Surgery, University of California Irvine, 3800 W. Chapman Ave. Suite 6200, Orange, CA 92868 USA

**Keywords:** Breast cancer, Oncofertility, Fertility counseling, Quality of life, Cryopreservation

## Abstract

**Introduction:**

Treatment-related infertility remains a paramount concern for young breast cancer patients. While emphasis on oncofertility is increasing, provision of fertility preservation remains suboptimal. This study evaluated oncofertility counseling (OC) and referral patterns to identify barriers to fertility preservation in this population.

**Methods:**

A single-institution retrospective analysis was performed of women aged 40 years and younger with breast cancer from 2014 to 2024. Patient demographics, tumor and treatment characteristics, OC, referral to a reproductive endocrinologist (REI), and childbearing were evaluated.

**Results:**

Of 145 patients, 80 (55.2%) received OC, while 65 (44.8%) did not. Of those who received OC, 98.7% were counseled by their oncologist, compared with 23.4% who were counseled by their breast surgeon. Twenty-five women (31.7%) who received OC were referred to an REI, and 7 (29.2%) achieved childbearing. Patients who received OC were younger (35 vs. 38 years; *p* < 0.001) and nulliparous (45.0% vs. 18.5%; *p* = 0.001). Patients with advanced disease and who received neoadjuvant chemotherapy were more likely to receive OC (both *p* < 0.05). There was no difference in disease recurrence or survival between patients who received OC or referral to REI (*p* > 0.05).

**Conclusion:**

OC is disproportionately provided to younger, nulliparous patients with advanced disease; however, all young breast cancer patients can benefit from OC, given the impact of cytotoxic and endocrine therapies on reproductive health. Breast surgeons’ early role in the care of breast cancer patients offers an underutilized opportunity for OC. All providers should be prepared to discuss the impact of patients’ diagnoses on fertility to maximize patient education and access to fertility preservation.

Breast cancer is the most common cancer among women,^1^ and in the past 5 years there has been a rise in breast cancer diagnoses among patients of childbearing age.^2^ Many treatments for breast cancer, including chemotherapy and endocrine therapy, can negatively impact fertility for premenopausal women. As incidence rates of breast cancer increase among the young adult population, so does the risk of chemotherapy-related infertility.^3^ Premenopausal women, who are more likely to be diagnosed with aggressive forms of breast cancer, often undergo more intensive treatment protocols, further jeopardizing their reproductive health.^4^

Breast cancer often carries a favorable prognosis, especially with the advent of modern day therapies, with only a 2.3% lifetime risk of breast cancer mortality. Thus, many patients experience long-term survival.^1^ As a result, the lasting effects on quality of life, particularly regarding reproductive health, become an important measure of treatment success. Therefore, it is ever more critical to address the long-term effects of oncologic treatment on reproductive health, as a woman’s future fertility can affect her quality of life. The emotional distress of women with ovarian insufficiency secondary to treatment has been well documented.^5^ In psychosocial-focused studies, it has been revealed that female cancer patients who are not offered provisions to protect their future reproductive health are subject to a lower quality of life because of subsequent infertility.^6^ As a result of risks to fertility, women may opt not to pursue chemotherapy, may choose different chemotherapy regimens, avoid endocrine therapy, or shorten the duration of endocrine therapy.^7^ Oncologists and surgeons have the opportunity to educate their patients on the associated risks of cancer therapies and engage in shared decision making by initiating oncofertility counseling (OC) early in the care process.

The 2018 American Society of Clinical Oncology (ASCO) Clinical Practice Guideline on Fertility Preservation strongly recommends healthcare providers address infertility risks as early as possible before treatment begins and refer patients to reproductive specialists accordingly.^8^ Research shows that fertility preservation is an important priority to breast cancer patients, with nearly half of reproductive-age patients in one study having taken steps to reduce the risk of infertility.^9^ However, in another survey study, approximately 70% of women talked to their providers about fertility-related concerns but just over half felt their concerns were adequately addressed.^10^ This highlights the critical but underutilized role that early counseling and shared decision making in oncofertility play in improving care for the patient’s overall well-being and addressing this aspect of breast cancer patients’ treatment plan.

Despite the growing recognition of oncofertility as an important priority in patients’ breast cancer care, significant gaps remain in the delivery of fertility counseling and access to preservation options.^11,12^ The goal of this retrospective study was to evaluate OC and referral practices at our institution. We sought to identify and mitigate barriers to fertility preservation for premenopausal women by optimizing the provision of oncofertility services to this patient population.

## Methods

We performed a retrospective analysis of women aged 40 years and younger who were treated for breast cancer from 2014 to 2024 at a single, large, tertiary care academic institution. Inclusion criteria included females aged 18–40 years treated for invasive and non-invasive breast cancers who were recommended to undergo systemic therapy in the form of chemotherapy or endocrine therapy. Patients were excluded if they had incomplete records, had already undergone menopause, or had known sterility before their cancer diagnosis. Our institution’s Institutional Review Board approved this study.

Preoperative patient demographic information, including age at diagnosis, sex, race and ethnicity, payor source, relationship status, and parity was collected, along with clinicopathologic details such as disease histology, clinical and pathologic stage, hormone receptor status, primary disease site, and presence of a genetic mutation. Patient histology was classified according to the International Classification of Diseases for Oncology, 3rd Edition. Histology was classified as invasive ductal carcinoma (IDC), invasive lobular carcinoma (ILC), ductal carcinoma in situ (DCIS), or mixed ductal and lobular carcinoma. Patients with hormone receptor-negative DCIS, phyllodes tumors, or sarcomas were excluded from analysis due to the low likelihood of treatment with systemic therapy. Treatment information included the type of surgical intervention, performance of axillary staging or axillary lymph node dissection, chemotherapy, endocrine therapy administration, enrollment in a clinical trial, and time interval to initiation of treatment. Interval to initiation of treatment was assessed as the number of days from tissue diagnosis to the first date of chemotherapy or upfront surgical resection. Subgroup analysis was performed evaluating patients for whom systemic therapy was recommended, including both chemotherapy and endocrine therapy.

The primary outcome assessed was patient receipt of OC, which was stratified into counseling performed by an oncologist or surgeon. Secondary outcomes included referral to a reproductive endocrinology and infertility specialist (REI), subsequent achievement of childbearing, disease recurrence, and vital status at last contact. OC was defined as the discussion of oncofertility in the patient’s note. Discussion of ovarian suppression only, such as use of a gonadotropin-releasing hormone (GnRH) agonist, was not considered OC.

Descriptive statistics were performed for all variables, and Mann–Whitney U tests were used to compare categorical variables. Categorical data were reported as percentages, while continuous data were reported as median with interquartile range (IQR). All *p*-values were two-sided, with a statistical significance level of < 0.05. All statistical analyses were performed in Stata 18.0 (StataCorp LLC, College Station, TX, USA; 2023).

## Results

**Fig. 1:**
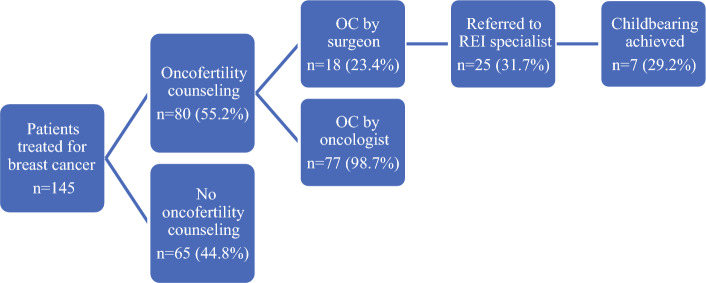
Patients who received oncofertility counseling, and resulting outcomes. *OC* oncofertility counseling, *REI* reproductive endocrinology and infertility specialist

A total of 145 women were identified as meeting the inclusion criteria, of whom 80 (55.2%) received OC and 65 (44.8%) did not (Fig. 1). Of those who did receive OC, 77 (98.7%) received counseling by their oncologist and 18 (23.4%) received counseling by their surgeon. Only one patient (1.3%) received counseling exclusively from their surgeon. Of the patients who received OC, 25 (31.7%) were referred to REI and 7 (29.2%) achieved subsequent childbearing.

When comparing demographic characteristics, patients who received OC presented at a younger median age than those who did not receive OC (35 vs. 38 years; *p* < 0.001) and were nulliparous (75.0% vs. 25.0%; *p* = 0.001) [Table 1]. Among those with children, only 45.4% received OC. There were no significant differences in relationship status, number of children, or presence of a genetic mutation between those who received OC and those who did not (all *p* > 0.05). There were no other significant differences in race, ethnicity, or payor status between those who did and did not receive OC (all *p* > 0.05).

**Table 1:** Demographic and oncologic characteristics of patients treated for breast cancer with and without oncofertility counseling

	Oncofertility counseling [*n* = 80]	No oncofertility counseling [*n* = 65]	*p*-Value
Age, years [median (IQR)]	35 (30.5–38)	38 (36–40)	<0.001
Race			0.55
White	58 (56.3)	45 (43.7)	
Black	4 (80.0)	1 (20.0)	
Asian	16 (50.0)	16 (50.0)	
Other	2 (40.0)	3 (60.0)	
Hispanic	29 (61.7)	18 (38.3)	0.27
Insurance			0.57
Medicaid	35 (58.3)	25 (41.7)	
Private insurance	45 (53.6)	39 (46.4)	
Childbearing status			0.001
Nulliparous	36 (75.0)	12 (25.0)	
Parous	44 (45.4)	53 (54.6)	
No. of children [median (IQR)]	2 (1–3)	2 (1–3)	0.48
Relationship status			0.16
Single	27 (64.3)	15 (35.7)	
Married	53 (51.5)	50 (48.5)	
Known genetic mutation	18 (64.3)	10 (35.7)	0.27

Data are expressed as *n* (%) unless otherwise specified

*IQR* interquartile range

Patients with IDC were more likely to receive OC than those who had ILC, and no patients with DCIS received OC (IDC 57.5% vs. ILC 33.3% vs. DCIS 0%; *p* < 0.001) [Table 2]. Those presenting with cT stage ≥ 2 were significantly more likely to receive OC than those not (61.0% vs. 39.0%; *p* = 0.04). Patients with clinically node-positive disease were also more likely to receive OC (61% vs. 33.9%; *p* = 0.006), as were those with clinical prognostic stage groups ≥ 2 (63.3% vs. 36.7%; *p* = 0.001). There were no significant differences in receipt of OC by tumor grade, hormone receptor, or HER2 status (all *p* > 0.05).

**Table 2:** Oncologic characteristics of patients treated for breast cancer with and without oncofertility counseling

	Oncofertility counseling [*n* = 80]	No oncofertility counseling [*n* = 65]	*p*-Value
Tumor histology			0.02
Ductal carcinoma in situ	0 (0)	6 (100)	
Invasive ductal carcinoma	77 (57.5)	57 (42.5)	
Invasive lobular carcinoma	1 (33.3)	2 (66.7)	
Mixed ductal and lobular carcinoma	2 (100)	0 (0)	
Tumor grade			0.40
1	4 (44.4)	5 (55.6)	
2	36 (56.3)	28 (43.8)	
3	36 (53.7)	31 (46.3)	
Hormone receptor status			0.75
Negative	19 (57.6)	14 (42.4)	
Positive	61 (54.5)	51 (45.5)	
HER2+	20 (51.3)	19 (48.7)	0.44
Triple negative	14 (66.7)	7 (33.3)	0.25
Clinical T stage			0.06
cT0	2 (100.0)	0 (0)	
cTis	2 (20.0)	8 (80.0)	
cT1mi/a	3 (75.0)	1 (25.0)	
cT1b	1 (20.0)	4 (80.0)	
cT1c	11 (45.8)	13 (54.2)	
cT2	35 (60.3)	23 (39.7)	
cT3	14 (53.9)	12 (46.2)	
cT4	12 (75.0)	4 (25.0)	
Clinically node-positive	41 (67.2)	20 (32.8)	0.01
Metastatic disease at presentation	8 (66.7)	4 (33.3)	0.40
Pathologic T stage			0.34
pT0	21 (65.5)	11 (34.4)	
pTis	3 (30.0)	7 (70.0)	
pT1mi/a	9 (45.0)	11 (55.0)	
pT1b	8 (44.4)	10 (55.6)	
pT1c	11 (61.1)	7 (38.9)	
pT2	17 (60.7)	11 (39.3)	
pT3	3 (75.0)	1 (25.0)	
pT4	1 (100)	0 (0)	
Pathologic node-positive	24 (61.5)	15 (38.5)	0.27
Mixed stage			0.01
0	2 (20.0)	8 (80.0)	
1	9 (34.6)	17 (65.4)	
2	42 (63.6)	24 (36.4)	
3	19 (61.3)	12 (38.7)	
4	8 (66.7)	4 (33.3)	

Data are expressed as *n* (%)

*HER2+* human epidermal growth factor receptor 2-positive

With regard to treatment characteristics, patients who received chemotherapy were significantly more likely to have received OC (59.1% vs. 40.9%; *p* = 0.005), with those who received neoadjuvant chemotherapy specifically having higher rates of OC (63.2% vs. 36.8%; *p* = 0.008) [Table 3]. There was no difference in receipt of OC among those who received adjuvant chemotherapy or those who received endocrine therapy (both *p* > 0.05). Patients who were enrolled in a clinical trial were also more likely to receive OC (78.1% vs. 21.9%; *p* = 0.01). There were no statistically significant differences in receipt of OC by type of mastectomy or axillary intervention performed (both *p* > 0.05). There was also no difference in time interval to initiation of treatment between those who received OC and those who did not. Additionally, among those who received OC and those who did not, there were no significant differences in disease recurrence or vital status at last contact, nor were there any differences in recurrence or vital status at last contact among patients referred to REI (all *p* > 0.05).

**Table 3:** Treatment characteristics and outcomes by oncofertility counseling

	Oncofertility counseling [*n* = 80]	No oncofertility counseling [*n* = 65]	*p*-Value
Mastectomy			0.63
Partial mastectomy	15 (50.0)	15 (50.0)	
Mastectomy	55 (55.0)	45 (45.0)	
Axillary surgery			0.26
Sentinel lymph node biopsy	48 (52.2)	44 (47.8)	
Axillary lymph node dissection	21 (63.6)	12 (36.4)	
Chemotherapy	68 (59.1)	47 (40.9)	0.005
Neoadjuvant chemotherapy [*n* = 95]	60 (63.2)	35 (36.8)	0.008
Adjuvant chemotherapy [*n* = 77]	45 (58.4)	32 (41.6)	0.29
Enrollment in a clinical trial	25 (78.1)	7 (21.9)	0.01
Endocrine therapy	55 (52.9)	49 (47.1)	0.49
Interval to treatment, days [median (IQR)]	48 (34.5–69.5)	46 (28–64)	0.07
Recurrence	5 (7.9)	7 (9.4)	0.78
Alive at last contact	74 (54.8)	61 (45.2)	0.75

Data are expressed as *n* (%) unless otherwise specified

## Discussion

This study highlights significant disparities in OC among reproductive-aged breast cancer patients at our tertiary academic medical center. Of all young women treated for breast cancer at our institution, about half received OC. Notably, the majority of these discussions occurred with the patient’s primary oncologist (98.7%), while breast surgeons played a more minor role (23.4%). The 2018 ASCO Clinical Practice Guideline on Fertility Preservation recommends all oncologic health providers be prepared to discuss infertility as a risk of cancer therapy as early as possible and refer all interested or ambivalent parties to a reproductive specialist before the initiation of treatment. Despite these guidelines, there remain significant disparities in the provision of OC to breast cancer patients of childbearing age. These findings underscore the need for improved multidisciplinary collaboration to ensure timely and equitable oncofertility care for all patients.

Our study demonstrated that about half of reproductive-aged breast cancer patients received OC with their healthcare providers, consistent with a widely reported range in OC rates from previous reported studies, ranging from 15 to 93%.^7,9,13,14^ Among Mayo Clinic patients, Mannion et al. demonstrated that 93% of breast cancer patients who were interested in future fertility received OC prior to initiating treatment.^9^ Another study from the Dana-Farber Cancer Institute found that of 657 reproductive-aged women with breast cancer surveyed, 72% discussed fertility concerns with their doctors.^11^ In contrast, Swain et al. performed a retrospective chart review of 306 reproductive-aged patients with breast cancer and found a significantly lower rate of OC at only 15%,^14^ while another retrospective study performed by McCray et al. from the Cleveland Clinic also noted a relatively low rate of OC of only 26%.^13^ Notably, our retrospective study demonstrated a 55.2% OC rate. While this is relatively higher than similar retrospective studies in the literature, it is still fails to address nearly half of the population. The significant variation in OC rates may reflect various biases, patient population, institutional protocols, and provider awareness, and also highlights a critical need to standardize delivery of OC.

Perhaps most surprising of our findings was the stark proportion of OC performed by oncologists compared with breast surgeons. While 98.7% of patients who received OC did so from their oncologist, only 23.4% of patients received OC from their surgeon. Furthermore, only one patient received exclusive counseling from her breast surgeon. This highlights a significant provider disparity and opportunity to improve the awareness and delivery of OC to these patients. Given the role of breast surgeons in the initial consultations of patients with breast cancer, surgeons have a unique opportunity to provide early counseling and referrals as recommended by ASCO guidelines. Plentiful and frequent oncofertility education, risk assessments, and shared decision making by multiple members of the multidisciplinary team may be beneficial to adequately address patients’ fertility concerns. Increasing conversations about oncofertility may be the first stepping stone to increasing provider and patient comfort with the complex decision-making process of balancing the risks and benefits of fertility preservation and patients’ oncology treatments.

We demonstrated that women with more advanced disease were more likely to receive OC, likely due to their increased need for systemic treatments that can impact fertility. However, even among patients who received chemotherapy, 40% still did not receive OC. Furthermore, although patients who received neoadjuvant chemotherapy were much more likely to receive OC, there were no differences among those receiving adjuvant chemotherapy. We suggest that this may be secondary to a higher provider and patient awareness at the time of diagnosis, initial consultations, and initiation of treatment. Similarly, there was no difference in OC receipt among those who received endocrine therapy, despite the effects of endocrine therapy on fertility. While the adverse effects of chemotherapy on ovarian health are well recognized, endocrine therapy carries its own risks that may be underrecognized and underaddressed. Endocrine therapy, such as tamoxifen and aromatase inhibitors, is often prescribed for 5–10 years, during which pregnancy is generally contraindicated due to potential teratogenicity and risk of recurrence.^15^ This prolonged treatment can delay childbearing into later years, when natural fertility declines. Younger women have been shown to have poorer compliance with endocrine therapy and are more likely to be non-adherent to endocrine therapy than older women, which may be partially attributed to by fertility concerns.^16^ However, emerging data, such as the POSITIVE trial, suggest that temporarily interrupting endocrine therapy for up to 2 years to allow pregnancy does not increase the short-term risk of cancer recurrence.^18^ Despite data supporting the safety of termporary endocrine therapy interruption on cancer outcomes, prior research has shown that women taking tamoxifen were significantly less likely to achieve child-bearing post-treatment compared with those not receiving endocrine therapy, despite no significant difference in ovarian reserve.^17^ These findings reinforce the need for timely, individualized OC to all reproductive-aged patients, regardless of presentation, to ensure they understand the risks to fertility, as well as available options to safely pursue childbearing.

While the early initiation of OC and referral to reproductive specialists is recommended, even those who do not receive upfront systemic therapy should be considered for OC as they may receive adjuvant chemotherapy or endocrine therapy, which may also have effects on patient fertility. As a result, patient fertility goals and the opportunity for OC should be continually considered and reassessed throughout the course of the patient’s treatment as they encounter additional systemic treatments that may affect their reproductive health.

Our data also showed that younger women without children were more likely to receive OC than their older or parous counterparts. Previous studies have shown similar findings, including a population-based study in France by Martinet et al., who found that younger age and nulliparity were significant factors influencing OC.^19^ Furthermore, Goodman et. al. reported nulliparous women and women under 35 years of age had higher odds of referrals for fertility preservation counseling.^20^ This suggests potential biases in counseling practices, where providers may make assumptions about family planning priorities based on a patient’s age, which may overlook older individuals’ preferences.

This study has some important limitations. As a retrospective chart review, there is the possibility of oncofertility discussions between patients and providers not documented in the electronic medical record. Patient fertility goals, including their perspectives and desires to preserve their fertility, were also not able to be assessed through the medical record review, which can impact whether a patient receives OC. Furthermore, the retrospective nature of this study fails to Additionally as a single-institution study, the data are limited to that received at our institution, which may lead to underreporting of REI referrals and fertility treatments and may also limit the generalizability of the findings. Patients lost to follow-up were additionally not able to provide data regarding childbearing or vital status. Finally, the study population was limited to women aged 18–40 years, which may prematurely exclude OC for older premenopausal breast cancer patients. Despite these limitations, the strength of this study remains in highlighting the need for more widespread and standardized OC, and highlighting potential biases and obstacles to routine oncofertility care for breast cancer patients of reproductive age.

## Conclusion

OC is a crucial component of treatment planning for reproductive-aged women diagnosed with breast cancer. All women of reproductive age, regardless of age, parity, or disease stage should be counseled by their healthcare providers on the potential impact of breast cancer treatment and fertility preservation options to maximize patient education, support, and access to fertility preservation. Further areas of research are needed to determine existing barriers to OC and to develop strategies to more effectively integrate oncofertility discussions into routine breast cancer care.
